# Case Report: Novel pathogenic variant in autosomal recessive *WNT10A*-related odonto-onycho-dermal dysplasia

**DOI:** 10.3389/fgene.2026.1750692

**Published:** 2026-03-27

**Authors:** Marianna Kalaszi, Ciaran Moore, Chrysoula Koniari, Theodoros Mavridis

**Affiliations:** 1 Department of Neurology, Tallaght University Hospital, Dublin, Ireland; 2 Division of Restorative Dentistry and Periodontology, Dublin Dental University Hospital, Dublin, Ireland; 3 Neurogenetics Unit, 1st Department of Neurology, Eginition Hospital, Medical School, National and Kapodistrian University of Athens, Athens, Greece

**Keywords:** autosomal recessive, ectodermal dysplasia, novel variant, odonto-onycho-dermal dysplasia, Wnt10a

## Abstract

Odonto-onycho-dermal dysplasia (OODD) is a rare, autosomal recessive disorder caused by pathogenic variants in the *WNT10A* gene, which plays a crucial role in ectodermal tissue development, including teeth, hair, nails, and skin. We describe an 18-year-old male of Afghan origin, born to consanguineous parents, who was evaluated during an extensive genetic family screening for an unrelated ataxia variant. The patient reported long-standing dental abnormalities, sparse hair, nail defects and hyperhidrosis. Examination revealed severe hypodontia, atypical dental morphology, retained deciduous teeth, smooth tongue due to reduced lingual papillae and dysmorphic nail growth. Trio whole-genome sequencing identified a novel homozygous frameshift variant in *WNT10A* [c.694del, p. (Arg232GlyfsTer11)] in the index patient. Segregation within the trio confirmed heterozygosity in both parents, supporting the diagnosis of OODD. Our case expands the spectrum of *WNT10A* mutations causing OODD by identifying a novel causative variant and highlights the value of comprehensive genetic screening, especially in populations with high consanguinity for assessing incidental clinically relevant findings and diagnosing rare conditions.

## Introduction

Odonto-onycho-dermal dysplasia (OODD) is a rare autosomal recessive form of ectodermal dysplasia caused by pathogenic mutations in the *WNT10A* (wingless-type MMTV integration site family member 10A) gene located in chromosomal region 2q35 (Omim.org, 2019). WNT10A protein belongs to the WNT (Wingless and INT-1) signaling pathway which plays a crucial role in tissue homeostasis and organ development by modulating proliferation, cell survival and cell behaviour (Doolan et al., 2021). In particular, WNT10A protein is involved in the regulation of ectodermal development, including dental morphogenesis, hair and nail growth (Doolan et al., 2021; Benard and Hammerschmidt, 2025). *WNT10A* mutations have been described to cause various presentations, including agenesis of permanent teeth and ectodermal dysplasia syndromes, such as odonto-onycho-dermal dysplasia (OODD) and Schöpf–Schulz–Passarge syndrome (SSPS) (Xu et al., 2017). OODD is characterized by abnormal dentition (hypodontia, microdontia and taurodontism), dry and thin hair, defective nail growth, palmomental hyperkeratosis, sweating abnormalities, lingual reduction of fungiform and filiform papillae causing smooth tongue appearance (Doolan et al., 2021; Benard and Hammerschmidt, 2025).

Herein, we report the case of a young male presenting with odonto-onycho-dermal dysplasia due to a novel pathogenic *WNT10A* variant.

## Case presentation

We present the case of an 18-year-old male patient who attended our Neurology Department for an extensive genetic family screening for a novel spastic ataxia variant identified in their first-degree cousins. At presentation, the patient reported a history of long-standing dental abnormalities, coarse and dry hair, and dystrophic nail growth. Additionally, the patient reported a history of delayed dental development–their first tooth erupted aged one while there was long-term retention of some of their deciduous teeth and non-eruption of most of their permanent teeth, as well. As a result, the patient was fitted with upper and lower removable dentures.

**FIGURE 1:**
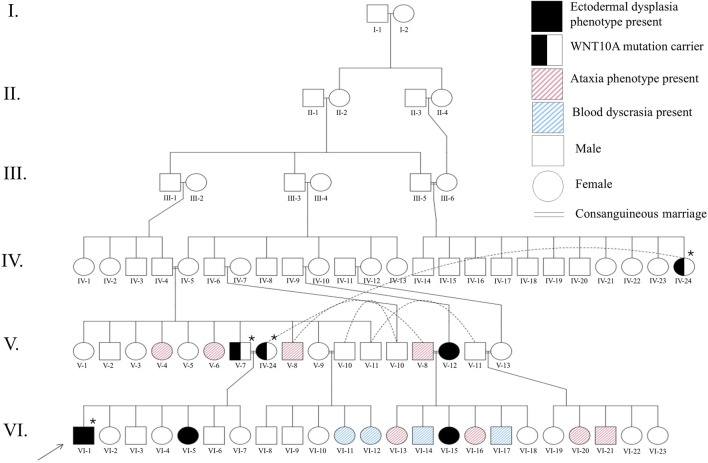
Family pedigree. Arrow shows the proband (VI-1). Several consanguinity loops are shown. Individuals with OODD, ataxia and blood dyscrasia phenotypes are marked. Study participants were represented with asterisk (*). Pedigree drawing was performed with QuickPed (Vigeland, 2022).

The patient was of Afghan origin and relocated to Ireland at the age of two. They were born to consanguineous parents, with a family history notable for multiple consanguineous marriages extending back to their great-grandparents (Figure 1). The patient has 6 siblings (four sisters and two brothers). One of the sisters (age 11) also has similar abnormalities in dental, hair and nail development. The fourth child died at 3 months of age due to congenital heart disease. They reported multiple family members living in Afghanistan with gait and mobility disturbances as well as blood dyscrasia (Figure 1). The patient was born prematurely at 37 weeks of gestation via normal delivery. There were no complications during the pregnancy or delivery, and no special care was required at birth. There was no delay in mental or physical developmental milestones. There were no other diagnosed medical conditions, and they did not take any medications. General physical, cardiovascular and neurological examinations were unremarkable.

**FIGURE 2:**
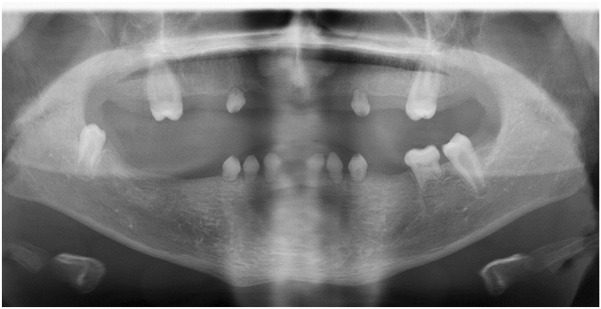
Panoramic radiograph aged 14 years old demonstrating severe hypodontia, multiple retained deciduous teeth, and atypical dental morphology.

**FIGURE 3:**
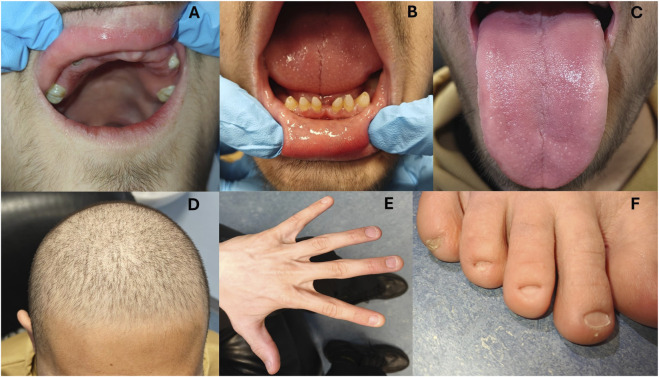
Upper panel: **(A)** Hypodontia. **(B)** Retention of some deciduous teeth. **(C)** Smooth tongue surface. Lower panel: **(D)** Thin, sparse hair. **(E)** Dysmorphic hand nail structure. **(F)** Absence of 3rd and 4th toenails.

On oral examination, the following permanent teeth were congenitally absent: the maxillary and mandibular central and lateral incisors, the maxillary and mandibular canines, the maxillary and mandibular first and second premolars, the maxillary second molars, and the maxillary and mandibular third molars. Unfortunately, the mandibular right first permanent molar had been lost due to dental caries. The crowns of the erupted maxillary first permanent molar teeth were microdont morphologically. The patient also had multiple retained deciduous teeth which all exhibited evidence of advanced root resorption radiographically. The maxillary right permanent first molar and mandibular permanent second molars were single rooted, and the mandibular left first permanent molar also had evidence of significant root resorption radiographically (Figures 2, 3A,B). The tongue surface was smooth with reduction of lingual papillae (Figure 3C). There was no cleft lip or palate. No dysmorphic facial feature was noted. Examination revealed abnormal, thin and sparse hair texture, most pronounced in the area close to his forehead (Figure 3D). Facial and body hair had normal development according to his age. Skin texture was normal to palpation and inspection, but the patient reported hyperhidrosis of the palms, soles, perinasal area and back. They exhibited thin, fragile and hypoplastic fingernails and toenails, with complete absence of bilateral 3^rd^ and 4^th^ toenails, as depicted in Figures 3E,F.

In the light of teeth, skin and nail abnormalities, a hereditary ectodermal dysplasia syndrome was suspected. The patient and both parents underwent trio whole-genome sequencing (Tri-WGS) at Centogene to investigate a potential genetic cause of the patient’s symptoms and to assess their carrier status for the ataxia-associated variant identified in their cousins. For this purpose, genomic DNA was extracted and underwent controlled enzymatic fragmentation followed by ligation of Illumina-compatible adapter sequences to construct paired-end sequencing libraries. Whole-genome sequencing was performed on an Illumina platform, generating approximately 30 × mean coverage across the nuclear genome. Resulting reads were aligned to the Genome Reference Consortium Human Build 37 (GRCh37/hg19) using standard alignment algorithms. Downstream processing and variant calling incorporated Illumina’s DRAGEN bioinformatics pipeline, complemented by CENTOGENE’s validated in-house analytical workflow.

The patient was found to carry a novel, homozygous pathogenic frameshift variant in *WNT10A,* (NM_025216.2): c.694del, p. (Arg232GlyfsTer11) (Richards et al., 2015). At this point, the diagnosis of *WNT10A*-related odonto-onycho-dermal dysplasia was established. This previously unreported variant is predicted to cause a frameshift, beginning at codon 232 in exon 3, resulting in an altered downstream amino-acid sequence and ultimately a non-functional WNT10A protein. Exon 3 is considered a mutational hotspot, as it encodes a key functional domain essential for proper activity of the WNT signaling pathway (Tardieu et al., 2017). Both parents were confirmed to be heterozygous carriers of the causative variant. Upon dental assessment, none of them showed signs of dental abnormalities, despite the fact that heterozygotes and particularly males, are known to display hypodontia (Bohrin et al., 2009).

The ataxia variant was not identified neither in our proband nor in their parents. Our patient and their mother were also heterozygous for a known pathogenic frameshift mutation in the *HBB* gene [c.17_18 del p. (Prof6ArgfsTer17)], which may lead to β-thalassemia of variable severity (Murad et al., 2021). We suspect that this variant could play a role in the reported blood dyscrasia among multiple family members, however we could not assess these individuals.

The patient’s care was continued within a multidisciplinary framework. A dermatology assessment was requested and the patient’s regular dentist was also informed of the findings. The patient and their parents were referred for genetic counselling. Unfortunately, we could not offer genetic testing for the affected sibling as she is underage, however we recommended her general practitioner to refer her to the Paediatric Developmental and Genetic Team for targeted testing and cardiac screening. Other likely affected family members reside in other countries.

## Discussion and conclusion

Herein, we report a patient carrying a novel homozygous pathogenic variant in *WNT10A*, presenting with clinical features consistent with odonto-onycho-dermal dysplasia (OODD). *WNT10A* gene plays a crucial role in embryonic and postnatal tissue and organ development and has been described in both non-syndromic tooth agenesis and syndromic tooth agenesis (OODD, SSPS and Tooth agenesis, selective, 4 (STHAG4)), with variable phenotype based on genotype (homozygous, compound heterozygous and heterozygous mutations) (Benard and Hammerschmidt, 2025).

Interestingly, as described above both parents were heterozygous with normal dentation, even though it is reported that half of heterozygotes (53.8%) can have any ectodermal manifestation (mainly tooth and nail anomalies), with males having a higher frequency of tooth anomalies than females, indicating reduced and sex-biased penetrance (Bohrin et al., 2009). The most plausible explanation for the normal dentition observed in both parents is that the identified *WNT10A* variant functions as a dose-dependent, low-penetrance allele, thus in its monoallelic state, the functional impact remains below the threshold required to disrupt odontogenesis.

Genotype–phenotype correlation studies consistently demonstrate that biallelic *WNT10A* variants, particularly those involving nonsense or clearly missense changes, are associated with severe oligodontia or ectodermal dysplasia. On the other hand, heterozygous carriers frequently exhibit only mild dental anomalies or even entirely normal dentition, suggesting that a single functional allele is often sufficient to maintain normal dentition (Doolan et al., 2021; Liu et al., 2022; Song et al., 2013; Tardieu et al., 2017; van den Boogaard et al., 2012). Functional analyses further indicate that *WNT10A* missense variants produce a vast spectrum of effects on WNT/β-catenin signaling, ranging from near-normal to markedly reduced activity (Zeng et al., 2020; Yuan et al., 2017; Kantaputra et al., 2022). This supports a dose-dependent loss-of-function model in which different alleles confer varying degrees of residual signaling capacity. Moreover, data show that several *WNT10A* variants may be enriched in individuals with hypodontia or oligodontia but are also detected in unaffected controls, consistent with incomplete penetrance and the likely contribution of additional genetic or environmental modifiers (Doolan et al., 2021; Song et al., 2013; van den Boogaard et al., 2012; Dhamo et al., 2016).

In summary, these findings support the hypothesis that, in this family, the heterozygous state results in partial and sub-threshold functional impairment compatible with normal tooth development, whereas the biallelic state in the proband leads to a sufficient reduction in signaling activity to cause tooth agenesis.

Currently there is no curative treatment for OODD. Multidisciplinary supportive management remains the gold standard. Emerging novel dental biomaterials, oral rehabilitation strategies, stem cell and grafting might further improve outcomes in the near future (Schneider, 2022). Furthermore, recent advances in translational research have expanded therapeutic opportunities as for ectodermal dysplasia (Schneider, 2022). In particular, increasing attention has been directed toward hypohidrotic ectodermal dysplasia (XLHED), which is one of the most common forms. An ongoing Phase 2 clinical trial investigating prenatal intra-amniotic administration of ectodysplasin A (*EDA*) gene in children with this condition with promising results (Schneider, 2022; Schneider et al., 2023).

In conclusion, our case report expands the mutational spectrum of *WNT10A*-related OODD by identifying a novel causative variant and highlighting the importance of exploring other possible phenotypic presentations during extensive genetic screening, especially in the face of consanguinity. Lastly, timely genetic testing could not only serve as a tool for diagnosis but also as a preventive measure in regions with high rates of consanguineous marriages.

## Data Availability

The original contributions presented in the study are included in the article, further inquiries can be directed to the corresponding authors.
